# Mindfulness-Based Stress Reduction on breast cancer symptoms: systematic review and meta-analysis

**DOI:** 10.31744/einstein_journal/2018RW4383

**Published:** 2018-11-26

**Authors:** Flavia Del Castanhel, Rafaela Liberali

**Affiliations:** 1 Universidade Federal de Santa Catarina, Florianópolis, SC, Brazil.

**Keywords:** Meditation, Breast neoplasms, Stress, psychological, Psychosocial intervention, Quality of life, Meditação, Neoplasias da mama, Estresse psicológico, Intervenção psicológica, Qualidade de vida

## Abstract

Mindfulness-Based Stress Reduction practices increase the capacity for concentration and attention, and these practices are particularly effective for people with breast cancer. To analyze the effects of the application of Mindfulness-Based Stress Reduction on breast cancer symptoms. Systematic review and meta-analysis were carried out. To find suitable studies, the PubMed/ MEDLINE database was searched using the keywords “breast cancer” and “Mindfulness-Based Stress Reduction”. Studies included were published between 2013 and 2017, written in English and showed methodological quality through the PEDro scale (score greater than 3). They also presented empirical evidence, had an experimental study design (randomized or non-randomized), and had full text available. For the meta-analysis, we used a random-effects model, with standardized mean differences and 95% confidence intervals. Seven studies were included, one non-randomized and containing only an intervention group of Mindfulness-Based Stress Reduction, and six randomized including samples of two or three groups. The non-randomized study showed 6 points on the PEDro scale, the randomized studies of two groups 6 to 7 points and studies with three groups showed 7 points. In the meta-analysis of the two randomized studies, the results, although not significant, revealed a moderate effect for Mindfulness-Based Stress Reduction on the outcome of fatigue, with a mean difference of −0.42 (95%CI −0.92- −0.07; p=0.09). Mindfulness-Based Stress Reduction seems to be a promising alternative for treatment of this disease's symptoms.

## RESUMO

A prática da Redução de Estresse Baseada em *Mindfulness* aumenta a capacidade de concentração e de atenção, sendo particularmente eficaz para pessoas com câncer de mama. O objetivo deste estudo foi analisar os efeitos da Redução de Estresse Baseada em *Mindfulness* nos sintomas de câncer de mama. Revisão sistemática e metanálise, na qual, para encontrar os estudos adequados, foi feita busca, no banco de dados PubMed/MEDLINE, usando as palavras-chave “*breast cancer*” e “*mindfulness-based stress reduction*”. Foram selecionados estudos em inglês publicados entre 2013 e 2017. Os estudos foram avaliados de acordo com sua qualidade metodológica pela escala PEDro (pontuação superior a 3), estudos que apresentaram evidências empíricas, desenho experimental (randomizado e não randomizado) e que estavam disponíveis na íntegra. Para a metanálise, utilizou-se um modelo de efeitos aleatórios com diferenças de médias padronizadas e intervalos de confiança de 95%. Sete estudos foram incluídos, sendo um não randomizado, contendo apenas um grupo de intervenção da Redução de Estresse Baseada em *Mindfulness* e seis estudos randomizados, divididos em amostras de dois grupos ou de três grupos. Os estudos não randomizados apresentaram escala PEDro de 6 pontos e os estudos randomizados de dois (de 6 a 7 pontos) e três grupos (7 pontos). Na metanálise, o resultado dos dois estudos randomizados selecionados, apesar de não significativo, revelou um efeito moderado da Redução de Estresse Baseada em *Mindfulness* sobre o desfecho da fadiga, com diferença média de −0,42 (IC95% −0,92- −0,07; p=0,09). A Redução de Estresse Baseada em *Mindfulness* apresenta-se como alternativa promissora para tratamento dessa doença.

## INTRODUCTION

Breast cancer is a complex and heterogeneous disease in terms of histology, spread, therapeutic response and different clinical outcomes.^(^ ^1^ ^–^ ^3^ ^)^ Currently, this is the second most common type of cancer affecting women,^(^ ^4^ ^,^ ^5^ ^)^ accounting for 60% of the deaths in developed countries.^(^ ^6^ ^)^ The incidence rate is increasing globally, for example, in 2008 approximately 1.38 million new cases of breast cancer were diagnosed^(^ ^7^ ^)^ and 1.7 million in 2012,^(^ ^8^ ^,^ ^9^ ^)^ with estimation of 1.38 to 1.7 million new cases each year,^(^ ^10^ ^,^ ^11^ ^)^ which makes this chronic disease a major public health problem.^(^ ^12^ ^,^ ^13^ ^)^

This disease occurrence is attributed to hereditary and environmental factors.^(^ ^14^ ^)^ Low incidence rates have been observed in countries such as Africa and Asia, and high rates in Western and Northern Europe, Australia, New Zealand and North America.^(^ ^15^ ^)^ In addition, racial/ethnic disparities have been observed in African-American women who have 41% higher chance of dying from breast cancer than white women, although the disease has a higher incidence among white women.^(^ ^16^ ^)^

Breast cancer is characterized by negative aspects including physical, mental and psychological symptoms.^(^ ^17^ ^)^ The most prevalent psychological symptoms are stress, anxiety, depression and impaired cognitive function,^(^ ^18^ ^–^ ^22^ ^)^ as well as physical symptoms such as pain, sleep disturbances and fatigue,^(^ ^23^ ^–^ ^25^ ^)^ which can trigger fear of death, recurrence, altered body image, and diminished well-being, among others.^(^ ^26^ ^–^ ^29^ ^)^ Fatigue is defined as tiredness, exhaustion or lack of energy that leaves participants unmotivated, impairs well-being,^(^ ^30^ ^)^ and affects individuals during and after treatment between 40% and 80% of cases.^(^ ^31^ ^)^

Many patients with breast cancer turn to complementary therapies to deal with the symptoms of the disease.^(^ ^32^ ^,^ ^33^ ^)^ A total of 33% to 47% of women worldwide and 48% to 80% of American women make use of such therapies,^(^ ^34^ ^)^ and meditation is one of complementary alternatives that positively influences the rehabilitation by reducing pain, stress, anxiety, depression, fatigue, and even the side effects caused by drug treatments.^(^ ^35^ ^,^ ^36^ ^)^ Meditation was originally proposed by Kabat-Zinn and it has been successfully incorporated in a number of clinical interventions.^(^ ^37^ ^)^

Currently, there is a range of therapeutic approaches based on mindfulness, such as Mindfulness-Based Eating Awareness Training (MB-EAT), Mindfulness-Based Relationship Enhancement (MBRE), Mindfulness-Based Relapse Prevention (MBRP), Mindfulness-Based Cognitive Therapy (MBCT) and Mindfulness-Based Stress Reduction (MBSR).^(^ ^38^ ^)^ Mindfulness-Based Stress Reduction is a standard protocol that addresses multiple forms of mindfulness practice, with elements of *hatha yoga* added to it.^(^ ^39^ ^)^

Studies show many benefits of meditation for breast cancer patients, such as decreased stress and anxiety, improvement of psychosocial factors, sleep quality and life perspective and feelings of empowerment, competence, personal strength, sense of calm, serenity and balance.^(^ ^23^ ^,^ ^40^ ^)^

## OBJECTIVE

To analyze the effects of the practice of Mindfulness-Based Stress Reduction on symptoms as fatigue, depression, anxiety and cognitive aspects in women with breast cancer.

## METHODS

### Search strategy

Studies were identified using the US National Library of Medicine/National Institutes of Health/MEDLINE (NLM/NIH/MEDLINE) − PubMed database. The procedures related to searching the database complied with the following steps:

– **First step:** identification of the keyword controlled by the Medical Subject Headings (MeSH) “breast cancer” and the not controlled “MBSR mindfulness-based stress reduction”. These terms were searched in MEDLINE database: “breast neoplasms” OR “breast” AND “neoplasms” OR “breast neoplasms” OR “breast” AND “cancer” OR “breast cancer” AND “MBSR” AND “mindfulness” OR “mindfulness” AND “based” AND “Stress” OR “stress” AND reduction.
– **Second step:** two reviewers independently screened the reports in two phases as proposed by Cook et al.^(^ ^41^ ^)^ Phase one, articles that included the key terms were screened by their titles and abstracts for relevance. Then, in phase 2, the full texts of the relevant articles were retrieved to assess their eligibility.

### Inclusion and exclusion criteria

Inclusion criteria for articles were: written in English from the PubMed/PMC database; directly addressed the topic breast cancer and mindfulness-based stress reduction; that demonstrated empirical evidence, with experimental study design (randomized or non-randomized); available in full text; that obtained a score greater than 3 on the Physiotherapy Evidence Database (PEDro; http://www.pedro.org.au); and with the year of the study limited to between 2013 and 2017.

Exclusion criteria were: other types of cancer different from breast cancer; other therapies (with MBSR only accepted alone or in comparison with other therapies); other symptoms that were not fatigue, depression, anxiety and cognitive aspects; and not written in English language.

### Methodological quality

The methodological quality of all studies was assessed using the PEDro^(^ ^42^ ^)^ (Table 1). It is a free database of randomized controlled trials, systematic reviews and clinical practice guidelines in physiotherapy. PEDro is based on the Delphi list and its purpose is to help its users to identify more rapidly which of the known or suspected randomized clinical trials (*i.e*. randomized controlled trials or RCTs) available at PEDro database are likely to be internally valid (criteria 2 to 9), and could have sufficient statistical information to make their results interpretable (criteria 10 to 11).^(^ ^42^ ^)^

**Table 1:** Physiotherapy Evidence Database (PEDro)

All criteria	Yes/No	Score
Eligibility criteria were specified	Yes/No	1/0
Subjects were randomly allocated into groups (in a crossover study, subjects were randomly allocated in the order in which treatments were received)	Yes/No	1/0
Allocation was concealed	Yes/No	1/0
The groups were similar at baseline regarding the most important prognostic indicators	Yes/No	1/0
There was blinding of all subjects	Yes/No	1/0
There was blinding of all therapists who administered the therapy	Yes/No	1/0
There was blinding of all assessors who measured at least one key outcome	Yes/No	1/0
Measures of at least one key outcome were obtained from more than 85% of the subjects initially allocated to groups	Yes/No	1/0
All subjects for whom outcome measures were available received the treatment or control condition as allocated or, if this was not the case, data for at least one key outcome was analyzed by “intention to treat”	Yes/No	1/0
The results of between-group statistical comparisons are reported for at least one key outcome	Yes/No	1/0
The study provides both point measures and measures of variability for at least one key outcome	Yes/No	1/0
	Total	Total /11

Source: PEDro. Physiotherapy Evidence Database [Internet].

Australian: The center for evidence-based physiotherapy; 1999 [cited 2015 June 26]. Available from: http://www.pedro.org.au/wp-content/uploads/PEDro_scale.pdf ^(^ ^42^ ^)^

### Risk of bias assessment

Methodological quality was independently assessed by at least two reviewers using the Cochrane collaboration risk for bias tool that considered seven different domains: adequacy of sequence generation; allocation sequence concealment; blinding of participants and caregivers; blinding for outcome assessment; incomplete outcome data; selective outcome reporting; and the presence of other potential sources of bias not accounted for in the other six domains. The estimated overall risk of bias for each trial was categorized as low (if the risk of bias was low in all key domains), unclear (if there is low or unclear risk of bias for all key domains) or high (if the risk of bias was high in one or more key domains).^(^ ^43^ ^)^

### Data analysis

Meta-analysis was conducted with two studies classified as randomized, as they had scores of fatigue as continuous data. The Stata software (version 12.0; Stata Corp., College Station, USA) was used for data synthesis and analysis. Post-intervention intergroup effect sizes were calculated using a random effects model (as this allows generalization of findings beyond the set of included studies) and variability in the estimates, with 95% confidence intervals (95%CI), and stratified by type of comparison group (MBSR *versus* control).

Next, Hedges' (adjusted) g was used to calculate the effect size for each study. The magnitude of Hedges' g may be interpreted using Cohen's^(^ ^44^ ^)^ convention as small (0.2), medium (0.5), and large (0.8). To establish whether the results of the studies were consistent, tests of heterogeneity were performed, using Q and I^2^ statistics. Q statistics calculates the probability value for the heterogeneity of studies (significant heterogeneity is indicated by a p value ≤0.05). An I^2^ value of 0% indicates no observed heterogeneity, while values of 25%, 50%, and 75% are considered low, moderate, and high.^(^ ^45^ ^)^ Publication bias was assessed using a funnel plot.

## RESULTS

### Search results and study characteristics

**Figure 1:**
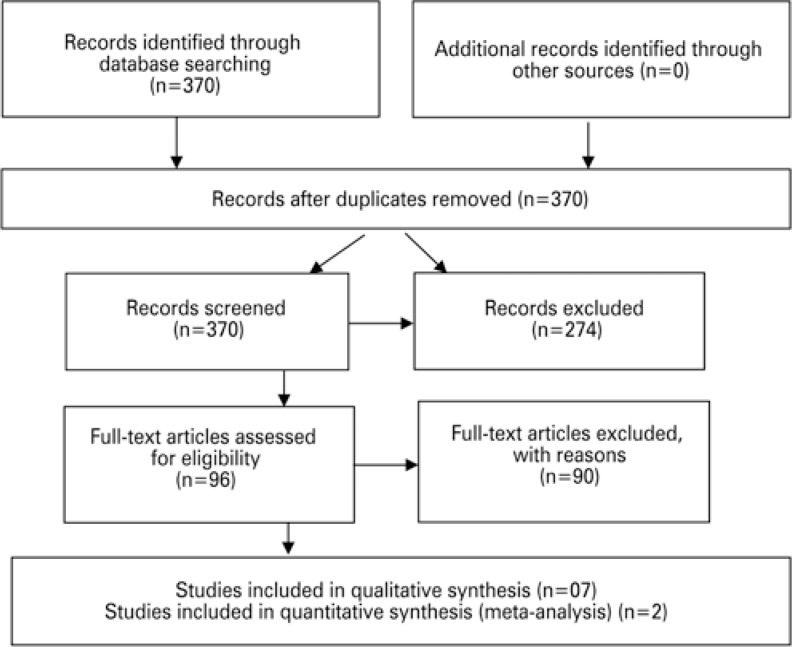
Flow diagram of search

The search located 370 studies in the MEDLINE (PubMed/PMC) database using the keywords “Mindfulness-Based Stress Reduction” AND “breast cancer”. Of these, seven studies were selected for analysis because they met the inclusion criteria (Figure 1).

For a description of the studies in the tables, the following categories were taken into account: the year of publication, local where the study was conducted, the classification in PEDro scale, the scales used as measuring instruments, the periodical of publication, the sample (age and sex), the intervention (mindfulness) monitoring of the sample and the results obtained.

The countries where the studies were developed were the United States [3; (42.8%)], Iran [2; (28.6%)], England [1; (14.3%)] and Sweden [1; (14.3%)]. The publication of the articles occurred in the years 2013 [1; (14.3%)], 2014 [3; (42.8%)], 2015 [2; (28.6%)] and 2017 [1; (14.3%)]. The articles used several scales for the analysis, and the most used scale was the European Organization for Research and Treatment of Cancer Quality of Life Questionnaire (EORTC-QLQ-30) (Table 2).

**Table 2:** Characteristics of the included studies

Studies	Studies local	Questionnaire
Henderson et al.,^(^ ^46^ ^)^	4 practice sites of Massachusetts, United States	SCL-90-R, DWI
Reich et al.,^(^ ^47^ ^)^	University of South Florida in Tampa, Florida, United States	MDASI
Rahmani et al.,^(^ ^48^ ^)^	Division of Oncology and Radiotherapy of Imam Hossein hospital in Tehran, Iran	EORTC QLQ-C30
Lengacher et al.,^(^ ^49^ ^)^	H. Lee Moffitt Cancer Center and Research Institute, in Tampa, Florida, United States	STAI; CAMSR
Eyles et al.,^(^ ^50^ ^)^	3 local oncology, United Kingdom	BFI; HADS
Rahmani et al.,^(^ ^51^ ^)^	Division of Oncology and Radiotherapy of Imam Hossein hospital in Tehran, Iran	FSS; EORTC QLQ-C30
Sarenmalm et al.,^(^ ^52^ ^)^	Research and Development Centre, Sweden	HADS

SCL-90-R: Symptom Checklist-90-Revised; DWI: Dealing with Illness Questionnaire; MDASI: MD Anderson Symptom Inventory; EORTC QLQ-C30: European Organization for Research and Treatment of Cancer Quality Life Questionnaire; STAI: State-Trait Anxiety Inventory; CAMSR: Cognitive and Affective Mindfulness Scale-Revised; BFI: Brief Fatigue Inventory; HADS: Hospital Anxiety and Depression Scale; FSS: Fatigue Severity Scale.

### Population characteristics

As described in table 3, the samples of the seven studies were composed only by women aged between 20 and 80 years, totaling 532 participants. All studies used MBSR as the intervention, based on the manual of Kabat-Zinn^(^ ^46^ ^–^ ^52^ ^)^ and performed with women diagnosed with breast cancer stage zero to III,^(^ ^47^ ^,^ ^49^ ^)^ phase I and II,^(^ ^46^ ^)^ phase I, II, III,^(^ ^48^ ^,^ ^51^ ^)^ and cancer with metastasis.^(^ ^50^ ^)^

**Table 3:** Characteristics of the studies interventions

Author	PEDro scale criteria	Subjects	Intervention	Findings
Henderson et al.,^(^ ^46^ ^)^	7/11	163 women with stage I or II of breast cancer (20 to 65 years old)	G1 = UC (n=58) G2 = MBSR (breast cancer) (n=53) G3 = NEP (n=52) Follow-up: 4 months	Anxiety - SCL-90-R mean (SD) p value Follow-up: 4 months MBSR *versus* UC = 0.14 (0.05) *versus* 0.28 (0.05); p≤0.05 Depression - SCL-90-R mean (SD) p value Follow-up: 4 months MBSR *versus* NEP = 0.31 (0.08) *versus* 0.58 (0.08); p≤0.05
Reich et al.,^(^ ^47^ ^)^	6/11	41 women with stage 0, I, II, or III of breast cancer (mean, SD) 58.2 (9.5) years old)	G1 = UC (n=24) + standard clinic visits G2 = MBSR* (breast cancer) 6-week program (n=17)	Fatigue - MADSI mean (SD) p value Baseline to 6 weeks UC = 9.1 (7.1) to 7.3 (6.0) ns MBSR = 8.8 (6.9) to 4.6 (5.3); p≤0.05 Cognitive/psychological - MADSI mean (SD) p value Baseline to 6 weeks UC = 9.1 (8.7) to 7.8 (8.5) ns MBSR = 7.1(10.1) to 4.3 (5.1) ns
Rahmani et al.,^(^ ^48^ ^)^	7/11	36 women with stages I, II, III of BC (38 to 49 years old)	G1= CG (n=12) G2= GTM (n=12) G1= MBSR (breast cancer) (n=12) Follow-up: 2 months	Fatigue - EORTC QLQ-C30 mean (SD) p value Baseline to 8 weeks to follow-up CG = 76.85 (5.72) to 71.29 (12.93)† to 72.22 (11.11); p≤0.00 GTM = 75.00 (8.37) to 64.81 (7.97)† to 76.85 (10.00); p≤0.00 MBSR = 77.77 (4.73) to 37.96 (8.81)† to 47.22 (11.72)‡; p≤0.00, baseline to 8 weeks†/8 weeks to follow-up‡ Cognitive/psychological - EORTC QLQ-C30 mean (SD) Baseline to 8 weeks to follow-up CG = 59.72 (8.58) to 59.72 (11.14) to 58.33 (15.07) GTM = 45.83 (10.36) to 61.11 (10.86) to 58.33 (8.71) MBSR = 62.50 (10.35) to 75.00 (11.23) to 72.22 (12.97)
Lengacher et al.,^(^ ^49^ ^)^	7/11	82 women with stages 0, I, II, III of breast cancer (mean 57 years old)	G1 = UC (n=42) waitlisted Control Group were offered the MBSR (breast cancer) G2 = MBSR (breast cancer) 6-week program (n=40) Follow-up: 2 week	Depression - mean (SD) Baseline to 6 weeks UC =14.2 (8.5) to 4.0 (1.7) MBSR = 13.3 (12.0) to 7.2 (4.6) Anxiety - mean (SD) Baseline to 6 weeks UC = 40.4 (11.9) to 6.4 (12.1)† MBSR = 35.3 (12.0) to 7.8 (9.8)†
Eyles et al.,^(^ ^50^ ^)^	6/11	19 women with metastatic breast cancer (37 to 65 years old)	G1 = MBSR = adapted (the class sessions were reduced to 2 hours in length (instead of 2 hours and 30 minutes) except the first and last, which lasted 2 hours and 30 minutes. The day of mindfulness in week 6 was 4 hours and 30 minutes (instead of 6-7 hours), and the mindfulness home practice using CDs of the above-mentioned mindfulness practices was 30 min/day, instead of 45 minutes/day) Follow-up: 24 week	Fatigue - BFI mean (SD) p value Baseline to 8 weeks = 4.19 (2.32) to 3.86 (2.45) ns 8 weeks to follow-up = 3.86 (2.45) to 3.28 (1.86) ns Baseline to follow-up = 4.19 (2.32) to 3.86 (2.45) ns Depression - HADS mean (SD) p value Baseline to 8 weeks = 5.74 (3.28) to 5.12 (4.46) ns 8 weeks to follow-up = 5.12 (4.46) to 3.79 (3.14) p≤0.04 Baseline to follow-up = 5.74 (3.28) to 3.79 (3.14) p≤0.04 Anxiety - HADS mean (SD) p value Baseline to 8 weeks = 9.42 (3.49) to 6.66 (3.63) 8 weeks to follow-up = 6.66 (3.63) to 5.79 (3.22) Baseline to follow-up = 9.42 (3.49) to 5.79 (3.22) p≤0.00
Rahmani et al.,^(^ ^51^ ^)^	7/11	24 women with stages I, II, III of BC (30 to 55 years old)	G1= CG (n=12) G2 = MBSR (breast cancer) + yoga (n=12) Follow-up: 2 months	Severe fatigue - FSS mean (SD) p value Baseline to 8 weeks to follow-up CG = 5.69 (1.58) to 5.60 (1.45) to 5.52 (3.07) ns MBSR = 5.85 (1.97) to 4.81 (2.60)* to 4.95 (2.61)†; p≤0.00, baseline to 8 weeks*/8 weeks to Follow-up† Fatigue - EORTC QLQ-C30 mean (SD) p value Baseline to 8 weeks to follow-up CG = 76.85 (5.72) to 71.29 (12.93) to 72.22 (11.11) ns MBSR = 77.77 (4.73) to 37.96 (8.81)* to 47.22 (11.72)†; p≤0.00, baseline to 8 weeks*/8 weeks to follow-up† Cognitive/psychological - EORTC QLQ-C30 mean (SD) Baseline to 8 weeks to follow-up CG = 59.72 (8.58) to 59.72 (11.14) to 61.11 (16.41) ns MBSR = 62.50 (10.35) to 75.00 (11.23) to 72.22 (12.97)
Sarenmalm et al.,^(^ ^52^ ^)^	7/11	166 women diagnosed with cancer at (34 to 80 years old)	G1 = active control (8 weeks self-instructing MBSR program), n=52 G2=MBSR (8 weeks self-instructing MBSR program + instructor and weekly group sessions), n=62 G3=non MBSR n=52	Anxiety - HADS mean (SD) p value MBSR = 6.5 (4.3) to 6.0 (3.9) ns Non MBSR = 4.8 (3.6) to 5.5 (4.1) ns Active control = 5.6 (3.9) to 5.1 (3.9) ns Depression - HADS mean (SD) p value MBSR = 4.3 (3.7) to 3.3 (3.3) p≤0.00 Non MBSR = 3.5 (3.5) to 3.8 (3.8) ns Active control = 3.4 (3.4) to 3.8 (3.8) ns

* MBSR: Kabat-Zinn's original 8-week program, weekly 2-hours sessions.^(^ ^39^ ^)^ The program includes three components: (a) educational materials and exercises related to meditation practices and the mind-body connection, (b) practice time and a CD on which verbal support for four meditative practices was recorded (sitting meditation, walking meditation, body scan, and gentle hatha yoga), and (c) opportunity for group discussion, including time to answer questions related to barriers with formal and informal practice. Participants were asked to spend 15 to 45 minutes daily outside of the group sessions for formal and informal practice and to record their daily practice in a diary. Formal mindfulness practice includes sitting meditation, walking meditation, body scan, and yoga practice. Informal mindfulness meditation practices, or mindfulness in everyday life, incorporate an awareness of pleasant and unpleasant events, and encourage awareness of routine activities;

† GTM: 8-week program. Introducing the model/identifying rumination periods (metacognition enhancement)/practicing techniques of increasing attention/completing. Attention training technique form/homework; practicing detached mindfulness/showing the postponing of rumination in an experimental way for modifying uncontrollable beliefs/practicing attention training technique; checking homework, examining the rumination, positive thoughts and activity level/examining and extensive application of detached mindfulness/continuing to the challenge with positive thoughts about rumination/examining activity levels and increasing time of contemplation to reaction (sinking in thought), identifying and preventing harmful coping behavior (e.g. sleep or drinking alcohol)/practicing attention training technique/homework).

‡ The nutrition education intervention, led by registered dieticians, was a group intervention focused on dietary change through education and group meal preparation. Practices followed the principles of social cognitive theory and patient-centered counseling. The nutrition education intervention was equivalent to the MBSR in terms of contact time and homework assignments but did not contain any meditation or yoga. G1: group 1; UC: usual care (Control Group); G2: group 2; MBSR: Mindfulness-Based Stress Reduction; G3: group 3; NEP: nutrition education intervention; SCL-90-R: Symptom Checklist-90-Revised; SD: standard deviation; MADSI: MD Anderson Symptom Inventory; ns: no significant; CG: Control Group; GTM: metacognition group therapy; EORTC QLQ-C30: European Organization for Research and Treatment of Cancer Quality Life Questionnaire; CD: compact disc; BFI: Brief Fatigue Inventory; HADS: Hospital Anxiety and Depression Scale; FSS: Fatigue Severity Scale.

### Designs of the studies

The interventions of the studies had different designs:

– Nonrandomized studies [1; (14.3%)], containing only one MBSR Group.^(^ ^50^ ^)^
– Randomized studies [5; (83%)], with the samples split into two groups, comparing the MBSR Group with a control or Usual Care Group (3; (42.8%)];^(^ ^47^ ^,^ ^49^ ^,^ ^51^ ^)^ and with samples divided into three groups, comparing the MBSR Group with a Control Group and a nutritional intervention group [1; (14.3%)],^(^ ^46^ ^)^ or with a Control Group and a Metacognitive Treatment Group (MTG) [1; (14.3%)],^(^ ^48^ ^)^ or with a Control Group and MBSR + instructor and weekly group sessions [1; (14.3%)].^(^ ^52^ ^)^

### Methodological quality results

PEDro ranged from 6 to 7 (mean total of all studies of 6.71; standard deviation of 0.48). Non-randomized study presented a PEDro scale score of 6 points;^(^ ^50^ ^)^ the randomized three group studies the score was between 6 and 7 points;^(^ ^47^ ^,^ ^49^ ^,^ ^51^ ^)^ and three group studies had 7 points.^(^ ^46^ ^,^ ^48^ ^,^ ^52^ ^)^

### Mindfulness-Based Stress Reduction outcomes

The non-randomized study of Eyles et al.,^(^ ^50^ ^)^ demonstrated, as a result of the practice of MBSR, significant improvements in the scores of depression and anxiety and a tendency for improvement in the fatigue score. The three randomized studies containing two intervention groups showed the result that 8 weeks of MBSR practice led to significant decrease of the scores for fatigue,^(^ ^47^ ^,^ ^51^ ^)^ anxiety,^(^ ^49^ ^)^ and cognitive aspects.^(^ ^51^ ^)^ The two randomized studies containing three intervention groups showed that the MBSR improved fatigue,^(^ ^48^ ^)^ depression,^(^ ^52^ ^)^ anxiety and depression.^(^ ^46^ ^)^

### Trial bias and quality of evidence

**Figure 2:**
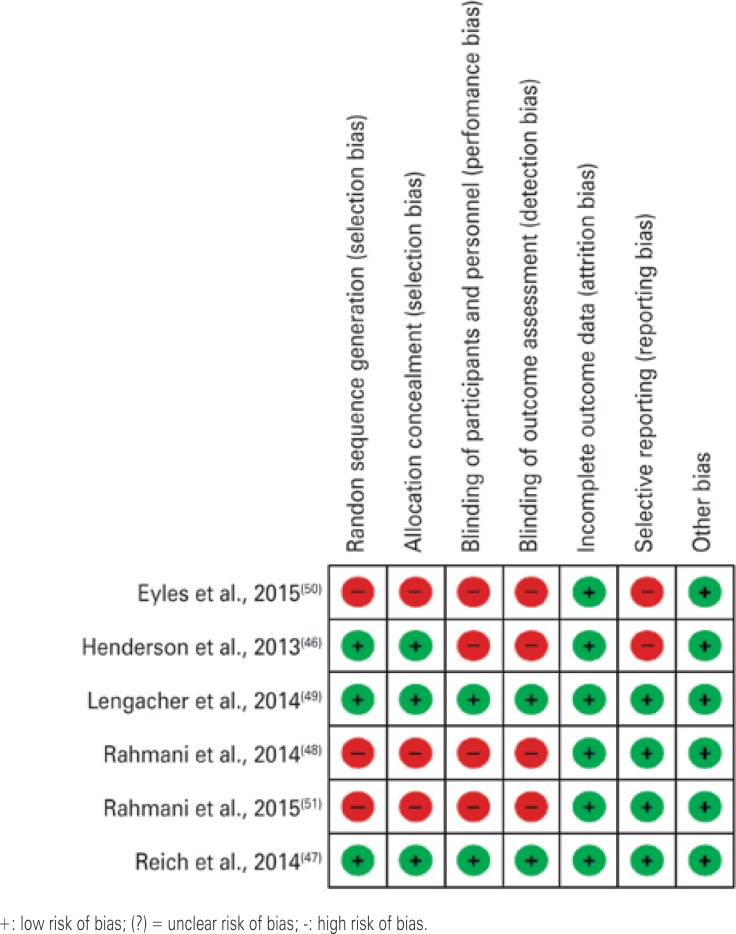
Methodological quality of trials using the Cochrane risk of bias tool

**Figure 3:**
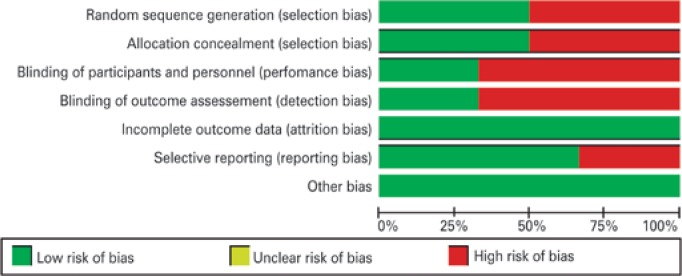
Overall risk of bias using the Cochrane risk of bias tool

The Cochrane risk of bias score for each citation is included in Figure 2. Only one of the six studies has low overall Cochrane risk of bias score (Figure 3). Because of the small number of trials included in this meta-analysis, we could not reliably examine funnel plots for publication bias.

### Meta-analysis results

Two studies presented continuous data, for a meta-analysis the results of which, although not significant, showed a moderate effect of MBSR on the outcome of fatigue with a mean difference of −0.42 (95%CI −0.92- −0.07; p=0.09). The forest plot shows the respective 95%CI for each study. It should be noted that the 95%CI of the articles and the diamond graph did not cross the line indicating statistically null results in favor of MBSR, regarding the scores of fatigue.

**Figure 4:**
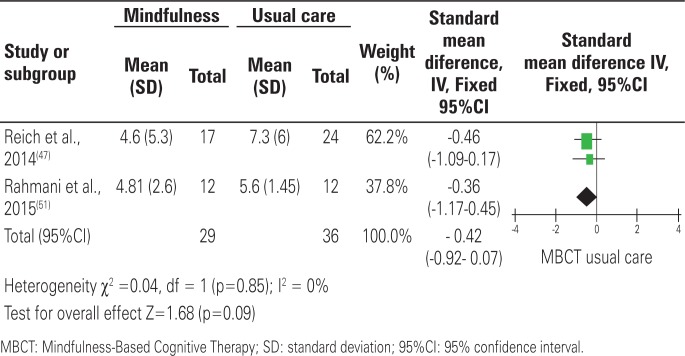
Forest plots of mindfulness-based stress reduction *versus* usual care (control) for fatigue

The result of the effect size (ES) of the MBSR regarding the scores of fatigue showed small ES for both studies, with the greatest effect in the study by Reich et al.,^(^ ^47^ ^)^ (0.46; 95%CI −1.09-0.17), followed by that of Rahmani et al.,^(^ ^51^ ^)^ (0.36; 95%CI −1.17-0.45), with no heterogeneity, according to the Q test analysis (p=0.85) and I^2^=0% (Figure 4).

## DISCUSSION

The results of the systematic review with meta-analysis (random effects model) and of the other studies (randomized and non-randomized studies) indicate that MBSR is likely to reduce the symptoms of breast cancer compared to other treatments (control, usual care, nutritional intervention, metacognitive treatment) by improving scores of depression, fatigue and cognitive aspects. It is assumed that, with the improvement of a breast cancer symptom, there is a tendency for the improvement of various other symptoms together.

With the improvement of a breast cancer symptom, there is a tendency for the improvement of various other symptoms together. In this review, some of these symptoms and their relationships with others were highlighted, so that we could understand the treatment of individuals with breast cancer.

Four studies showed MBSR as beneficial in decreasing the symptoms of fatigue.^(^ ^47^ ^,^ ^48^ ^,^ ^51^ ^)^ Fatigue is defined as the inability to initiate and maintain tasks that require attention and self-motivation,^(^ ^52^ ^–^ ^54^ ^)^ and is one of the most prevalent, severe and debilitating symptoms among cancer patients, leading to decreased physical functioning^(^ ^55^ ^)^ and becoming a major problem during breast cancer treatment.^(^ ^56^ ^)^

Armes et al.,^(^ ^57^ ^)^ highlighted that there is no pharmacological treatments for fatigue. However, interventions should focus on psychological, educational, social and group support therapies aiming to allow the individuals to interpret the fatigue, to respond to the symptoms with positive thinking and return to their daily activities.^(^ ^57^ ^,^ ^58^ ^)^ The practice of meditation appears to be beneficial in the treatment of fatigue, as it is considered a psycho-educational therapy^(^ ^59^ ^)^ that combines cognition with intensive meditation training,^(^ ^38^ ^)^ focusing on observation and working on cognitive and affective processes in order to teach the individuals to become more aware and to relate to themselves.^(^ ^60^ ^)^

The positive relationship between the practice of meditation and symptoms of breast cancer is consistent with several other studies. Some show reduced fatigue and increased physical vigor, such as the study of Carlson et al.,^(^ ^61^ ^)^ with 89 subjects of both genders and various types of cancer, in 6 weeks of meditation practice, and the study of Carlson et al.,^(^ ^62^ ^)^ with 63 individuals of both genders with cancer (breast cancer, ovarian, lymphoma and prostate), after 8 weeks of MBSR practice. Others show a reduction of fatigue with improved physical energy, such as the study of Carlson et al.,^(^ ^63^ ^)^ with 49 women with breast cancer and 10 men with prostate cancer, in 8 weeks of practice of MBSR, and Lengacher et al.,^(^ ^64^ ^)^ with 41 women with BC, after 6 weeks of MBSR practice.

Cognitive aspects also showed improvement with the practice of MBSR,^(^ ^48^ ^,^ ^51^ ^)^ as well as for the metacognition group therapy.^(^ ^48^ ^)^ Between 17% and 75% of patients with breast cancer, who perform chemotherapy, showed altered brain structure and function which suggests a pattern of diffuse brain injury that underlies the cognitive *deficits* that may occur during the first 6 months, followed by a recovery of 1 to 2 years and/or a period of stabilization.^(^ ^65^ ^)^ Executive functions (*e.g*., working memory) and processing speed, cognitive processes largely controlled by frontally mediated brain systems, have been most prominently reported.^(^ ^66^ ^)^

The memory has the function of storing and manipulating information for a short period of time, requiring integration of the prefrontal cortex with the other cerebral connections.^(^ ^67^ ^)^ Attention is a key modulator of cognitive processing, enabling us to select task-relevant stimuli and inhibit irrelevant information, sustain focus on cognitive performance, divide attention, and stay vigilant when needed.^(^ ^68^ ^)^ Chemotherapy can affect memory (verbal and visual), attention, concentration, multiple decision-making tasks, mental flexibility and speed of processing.^(^ ^69^ ^)^

Therefore, MBSR is a promising therapy as it is associated with increased density in the regions of the brain related to attention and sensory processing, including the prefrontal cortex and right anterior insula, leading to increased brain activation involved in the processing of emotions, sense of well-being and reduction of relapse and recurrence.^(^ ^70^ ^,^ ^71^ ^)^ A two-component model of mindfulness, involving self-regulation of attention (maintained on immediate experience) and attitudinal orientation (curiosity, openness, and acceptance).^(^ ^68^ ^)^

The study of Schellekens et al.,^(^ ^72^ ^)^ was consistent in showing the atmosphere in the MBSR training experienced by 37 women with breast cancer as safe and provide a context in which participants could connect with and trust one another and encourage patients to fell accepted, and helped them to facilitate each other learning processes, such as acknowledge their emotions and gaining different perspectives.

The metacognition group therapy also showed promising for the improvement of cognitive aspects in breast cancer. Metacognition group therapy is similar to MBSR, as it is based on the premise that the negative evaluation of disease (*i.e*., negative thoughts about cancer and its consequences) instigates and maintains anguish and, therefore, uses elements of mindfulness in the pursuit of self-knowledge, and understanding of feelings and emotions, focusing on interrupting negative thoughts.^(^ ^73^ ^,^ ^74^ ^)^

Studies have shown that MBSR improved depression and anxiety,^(^ ^46^ ^,^ ^49^ ^,^ ^50^ ^,^ ^52^ ^)^ as well as depression for the nutritional education intervention.^(^ ^46^ ^)^ From 22% to 50% of women with breast cancer have depression, 3% to 19% post-traumatic stress and 33% stress.^(^ ^75^ ^)^ Huang et al.,^(^ ^76^ ^)^ highlighted that almost 60% of breast cancer patients report high levels of anxiety, while 25.6 to 58% report living with depression.

Mindfulness is important to combat these psychological symptoms, as it leads the person to accept their inner experiences without judgment, reduce the usual or automatic responses to stressful experiences and develop a view of their life events (that cannot be changed), therefore, reducing the stress and psychological symptoms.^(^ ^77^ ^)^ Some meta-analyzes confirm that the practice of MBSR can help reduce psychological symptoms, such as mood and sleep disorders, depression and anxiety in women with breast cancer.^(^ ^9^ ^,^ ^59^ ^,^ ^78^ ^)^ Other randomized studies are consistent in showing the relationship of the practice of meditation and the improvement in the symptoms of depression and anxiety, with 6^(^ ^61^ ^,^ ^64^ ^)^ or 8 weeks of practice.^(^ ^62^ ^,^ ^63^ ^)^

The nutritional education intervention also showed an improvement in depression, with the use of similar elements to MBSR, in terms of group therapy held in 8 sessions with homework assignments (containing no meditation or yoga elements), but focused on nutritional education with dietary change through group meal preparation, following the principles of social cognitive theory and patient-centered counseling.^(^ ^46^ ^,^ ^79^ ^)^ Nutritional education intervention is important, as an intervention in the lifestyle, to reduce dietary fat intake, with modest influence on body weight, and it may improve the disease-free survival of patients with breast cancer.^(^ ^80^ ^)^

### Limitations

Some limitations of this meta-analysis should be considered. One is the quality of the studies, because not all achieve the maximum score on PEDro scale. In addition, some studies did not report basic data of descriptive and inferential statistics, providing only frequency values. Other limitation is attributed to the inclusion of non-randomized clinical trials without performing a meta-analysis of them. This choice was because of the need to observe in the literature articles to supply innovative results although not performed randomly. Another limit is the choice of the articles from just one database that can lead to lack of intervention studies with excellent quality available in other databases. This choice was made because this database is freely accessible and presents quality in its publications.

## CONCLUSION

This systematic review and meta-analysis suggests that Mindfulness-Based Stress Reduction can be considered a promising alternative for the treatment of breast cancer symptoms. The results show that Mindfulness-Based Stress Reduction, alone or integrated with other cognitive interventions (nutrition education intervention, Metacognition group therapy), is effective for main symptoms of breast cancer. These findings demonstrate the need for greater provision of alternative treatments for breast cancer symptoms, as this would reduce costs of the intervention and complement the usual treatment.
